# Using Virtual Reality Environments to Augment Cognitive Behavioral Therapy for Fears and Phobias in Autistic Adults

**DOI:** 10.1089/aut.2018.0019

**Published:** 2019-04-13

**Authors:** Morag Maskey, Jacqui Rodgers, Barry Ingham, Mark Freeston, Gemma Evans, Marie Labus, Jeremy R. Parr

**Affiliations:** ^1^ Institute of Neuroscience, Newcastle University, Newcastle upon Tyne, United Kingdom.; ^2^ Northumberland, Tyne and Wear NHS Foundation Trust, Newcastle upon Tyne, United Kingdom.; ^3^ Business Development and Enterprise, Faculty of Medical Sciences, Newcastle University, Newcastle upon Tyne, United Kingdom.

**Keywords:** autism, ASD, adults, anxiety, fear, phobia, virtual reality, cognitive behavior therapy

## Abstract

Fears and phobias are common in people on the autism spectrum and can impact on their ability to undertake usual daily activities. Graded exposure to the anxiety-provoking stimulus is a recognized method of treatment for fears/phobias in the nonautistic population but may pose specific difficulties for autistic people. For example, real-life exposure can be too anxiety-provoking to allow treatment to take place, and imaginal exposure can be problematic. To address this, we developed an intervention that combines cognitive behavioral therapy (CBT) with immersive virtual reality (VR) exposure to reduce anxiety. Following successful trials of this intervention with young people on the autism spectrum, we report a pilot study using the same intervention with autistic adults. Eight adults (aged 18–57 years) received one psychoeducation session and then four 20-minute sessions of graded exposure with a therapist in an immersive VR room (known as the Blue Room). Each participant completed all sessions showing that the intervention is feasible and acceptable. Outcomes were monitored at 6 weeks and 6 months postintervention. Five of the eight participants were classified as intervention responders and at 6 months after the end of intervention were experiencing real-life functional improvements. These preliminary findings show that VR-graded exposure alongside CBT may be an effective treatment for autistic people with phobias.

**Lay Summary:**

Why was this study done?

Anxiety is common in autistic adults. For some people, fears and phobias regarding everyday objects and situations occur frequently affecting everyday life. The main method to treat fears and phobias for people without autism is gradual exposure to the situation that causes anxiety. However, this method may be challenging for people on the autism spectrum. We wanted to test a new method of treatment that uses cognitive behavioral therapy (CBT) delivered with gradual exposure in a fully immersive virtual reality (VR) environment.

What was the purpose of this study?

We have already delivered this treatment successfully with autistic children. We wanted to test if this treatment would work for autistic adults. Changing traditional psychological treatments, such as CBT, to make it more suitable for autistic people is recommended by the National Institute for Health and Care Excellence.

What did the researchers do?

We recruited eight autistic adults (aged 18–57 years) with a fear/phobia and their supporter (parent/friend/support worker). Each adult had one session with a therapist to learn anxiety management techniques. They then had four 20-minute sessions of graded exposure with a therapist in an immersive VR room (known as the Blue Room). Each participant had a computer-generated scene designed for their specific anxiety-provoking situation. After four sessions, the participant tried real-life exposure with their supporter. We measured progress at 6 weeks and 6 months after the last VR session.

What were the results of this study?

Each participant completed all four sessions. This shows that the intervention was possible to deliver and acceptable to autistic people and therapists. Participants completed assessments at 6 weeks and 6 months after the VR sessions. Five of the eight participants were “responders” to the intervention. This means that 6 months after the last VR session, they still had real-life day-to-day improvements in relation to their phobia.

What do these findings add to what was already known?

We had not delivered this intervention to autistic adults previously. The findings show that this VR intervention has the potential to be an effective treatment for anxiety in autistic adults.

What are the potential weaknesses in the study?

This is a small study and future work will be a larger trial of this treatment—comparing results from people who get the intervention with people who do not. We would also want to have an outcome assessor who did not know whether people had received the intervention or not.

How will these findings help autistic adults now or in the future?

This new intervention has the potential to help autistic adults manage their anxiety in stressful situations and therefore may improve their quality of life.

## Introduction

People on the autism spectrum frequently have coexisting conditions,^1^ including mental health difficulties.^2^ Around 50% of adults on the autism spectrum experience levels of anxiety that affect everyday life, highlighting the need for effective treatments.^3–5^ Fears and phobias are one specific anxiety subtype. In the general population, fears and phobias are one of the most common anxiety presentations (cross-national lifetime prevalence of 7.7%)^6^ and are characterized by an excessive and persistent fear of a specific object or situation that interferes with an individual's daily functioning.^7^ Despite their prevalence, fewer people seek treatment for phobias than any other anxiety conditions.^8^ Phobias are common in autistic people and relate to a wide range of specific situations or triggers (e.g., crowded public transport, a supermarket, insects, and animals).^9–11^ Phobias may be atypical or unusual compared with those experienced by neurotypical people^11^ and can have a major impact on the individual and those around them, affecting everyday activities, learning, and social inclusion.^12,13^ Interventions that are specifically adapted for people on the autism spectrum are required to effectively treat phobias.

## Theoretical Framework

Graded exposure is a well-established evidence-based treatment for phobias in the general population^14^; however, traditional graded exposure protocols may be ineffective for people on the autism spectrum. Graded exposure usually begins with the person receiving the treatment imagining the feared object/situation in an exposure hierarchy paradigm that gradually progresses to *in vivo* exposure. Autistic people often experience difficulties with imagination and abstract thinking, which can make producing and controlling imaginal scenes difficult.^15,16^ Additionally, steps toward real-world exposure may feel too anxiety-provoking due to the potential uncontrollability of the stimuli, as progression through the hierarchy is attempted in natural environments; this can lead to poor treatment compliance. One way to potentially address some of these limitations is through the use of an immersive virtual reality environment (VRE). A VRE enables the therapy team to recreate the phobic situation through computer-generated scenes,^12^ reducing the reliance on imaginative and abstract thinking skills. The VRE also allows the exposure to be highly controllable and the anxiety hierarchy to be worked toward in a safe and calibrated manner. While in the VRE, participants can work with a therapist to practice techniques to manage their anxiety. Introducing cognitive behavioral therapy (CBT) techniques to manage anxiety (such as relaxation, anxiety recognition, and restructuring of thoughts), alongside gradual exposure to computer-generated images, may help maintain anxiety at manageable levels while the person stays in the situation and so allows habituation to occur. A parent/supporter can observe the treatment via a video link from an adjacent room. This intervention is compliant with existing recommendations from the UK National Institute for Health and Care Excellence (NICE) guidelines^17^ in relation to making adaptations to CBT to make it more effective for autistic people. Recommended adaptations include: a more concrete and structured approach with a greater use of written and visual information; involving a family member, partner, carer, or professional (if the autistic person agrees) to support the implementation of an intervention; maintaining the person's attention by offering regular breaks, and incorporating their special interests into therapy if possible (such as using computers to present information).

This article describes the next stage in our research program. We aimed to investigate the feasibility and acceptability of delivering this therapeutic approach to adults and its therapeutic potential. Our feasibility and acceptability aims were to: (1) explore whether autistic adults engage with the computer-generated VRE scenes in a way that allows them to approach and remain in the anxiety-provoking situation; (2) investigate participation and retention during the study; (3) explore whether the four treatment session structure (as used in Maskey et al.^12^ with children) was appropriate for autistic adults by using the same number of sessions and monitoring attendance and outcomes; (4) investigate whether the outcome measures used were sensitive to change; and (5) evaluate in a small case study design whether adults gained similar benefits to those seen in the children's study in terms of functional real-life outcomes following the intervention.

## Description of the Intervention

For this intervention, we used a unique immersive VRE known as the Blue Room. The Blue Room is an immersive technology using computer-generated images projected onto the walls and ceilings of a 360-degree seamless screened room. Participants are not required to wear a headset or goggles, and the therapist navigates with them through the scene using a handheld tablet computer. In the VRE, computer-generated scenes are individualized for each participant, incorporating an exposure hierarchy related to the feared stimulus, so that they can be gradually exposed to their phobia situation (e.g., going into a shop, getting onto a bus). While in the VRE, a therapist remains with the participant throughout the treatment session and supports them to utilize anxiety management strategies to keep anxiety at manageable levels and stay in the situation rather than leave if overwhelmed. We have demonstrated that four 20- to 30-minute sessions in the VRE led to reductions in anxiety in eight of the nine autistic children who presented with phobias.^12^ Following the intervention, these eight children were able to manage their anxiety situation; this improvement was sustained 12 months postintervention, for example, a child was able to travel on public transport. A further study involving 32 clinically referred autistic children randomized to receive the Blue Room intervention or delayed treatment also showed effectiveness for around half the children treated.^13^

## Methods

### Participants

The participants recruited were eight adults on the autism spectrum. Seven participants were recruited through a local National Health Service (NHS) adult autism diagnosis team. One participant was recruited through identification within a local autism support network. All participants had a confirmed autism spectrum diagnosis given by a multidisciplinary NHS team before recruitment.

To be included in the study, participants had to be interested in undertaking a short experimental intervention aiming to reduce phobia, aged between 18 and 60 years with a diagnosis of autism spectrum condition/autism/Asperger's syndrome, and considered by the referral and research team to have sufficient verbal skills to take part in the intervention. Participants needed at least one phobia that they identified as a treatment target. Participants also needed to identify an adult supporter (a family member, partner, friend, or formal carer/support worker) who could accompany them to the sessions in the Blue Room, observe the sessions in progress from an adjacent room, and then support the participant as they worked toward *in vivo* exposure to a real-life situation.

The exclusion criteria were adults with a severe coexisting mental health condition or functional impairment as indicated at initial screening, for example, severe depression; a history of severe conduct disorder and/or with severe generalized anxiety disorder; psychosis; substance abuse problem; and/or unable to travel to the virtual reality (VR) facility.

Eight adults on the autism spectrum participated (four men and four women; mean age 29.8 years, range 18.8–57.0 years). All participants were considered by the research team to have the cognitive ability to understand and follow the treatment approach; all used conversational speech. References to gender have been removed from any case descriptions to provide anonymity. Two participants lived independently and managed their own household, one participant was living with a partner, and five participants lived with their parents. All participants completed four treatment sessions in the VRE.

### Baseline measures

Measurement of autism spectrum characteristics at baseline was evaluated using the SRS-2 (Social Responsiveness Scale-second edition). The SRS-2 self-report version is a standardized questionnaire used to rate the social communication difficulties of autistic adults. The questionnaire takes around 15–20 minutes to complete, identifies social impairment associated with the autism spectrum, and quantifies its severity. A score of 66–75 indicates moderate impairment in social communication and a score of 76 or higher indicates severe impairment. In addition to a total score reflecting the severity of social deficits in the autism spectrum, five treatment subscale scores are provided: Social Awareness, Social Cognition, Social Communication, Social Motivation, and Restricted Interests and Repetitive Behavior. Participants' mean Social Responsiveness Questionnaire-2 (SRS-2) total score was 77.5 (range 69–90; SD 7.8) indicating a moderate-to-severe impairment in social communication.

### Outcome measures

Participation and retention measurement was measured by the number of VRE sessions completed for each participant and whether or not follow-ups were completed at 6 weeks and 6 months postintervention.

The following measures were completed preintervention, 6 weeks postintervention, and 6 months postintervention to allow measurement of impact of the intervention on behavior:

Target Behaviors were used to identify symptom change over time for the phobia targeted in the treatment. We have used this measure successfully in monitoring anxiety change previously.^12^ In a review of treatment of specific fears and phobias in children on the autism spectrum,^18^ target behaviors were the primary outcome measure in 10 of the 16 studies reviewed. The protocol used was developed by the Research Units on Pediatric Psychopharmacology (RUPP) Autism Network^19^ and was used previously in our VRE intervention studies with children. Having identified a specific anxiety target, questions such as “how often?” and “how distressed?” are asked in a standard interview format to the participant and their supporter. These interviews are then completed again at 6 weeks and 6 months postintervention. This enables a researcher to write a vignette describing the anxiety and its impact at three time points. In this study, the researcher collecting and writing the vignettes was not blind to intervention status. The 6 weeks and 6 months postintervention vignettes were then compared with those written before intervention by an expert panel (experienced researchers from our North East Autism Research group, who received training and practice in the rating technique) to assess degree of change. Panel participants were blinded to the time point the vignette related to. Each postintervention vignette was rated in comparison to the baseline vignette on a 9-point scale (from 1 “normalized,” 2 “markedly improved,” 3 “definitely improved,” 4 “equivocally improved,” 5 “no change,” 6 “equivocally worse,” 7 “definitely worse,” 8 “markedly worse” to 9 “disastrously worse”). This showed the degree of change from baseline. Each vignette pair was rated by four raters (individually, without discussion with others) and the mean rating recorded; a mean rating between 1 and 3 described a “treatment responder.” Arnold et al.^19^ reported an Intraclass correlation coefficient (ICC) of 0.895 for a panel of five experts. Following Arnold et al.,^19^ we have used the term “normalized” where the rating from the expert panel indicated that a participant was able to function without any impact of phobia.

To monitor anxiety, we used the Beck Anxiety Inventory (BAI) and the Generalized Anxiety Disorder 7 (GAD-7). The BAI is a 21-item, multiple choice self-report inventory measuring the severity of anxiety in adults and adolescents. The items in the BAI describe the emotional, physiological, and cognitive symptoms of anxiety. The BAI requires a basic reading level, can be used with individuals who have intellectual disabilities, and can be completed in 5–10 minutes. The GAD-7 is a self-report questionnaire for screening and severity measuring of generalized anxiety disorder. It has seven items, which measure severity of various signs of generalized anxiety disorder according to reported response categories with assigned points. Assessment is indicated by the total score.

To record symptoms of depression, we used the Patient Health Questionnaire-9 (PHQ-9). The PHQ-9 is a nine-item self-report depression scale of the Patient Health Questionnaire. The nine items of the PHQ-9 are based directly on the nine diagnostic criteria for major depressive disorder in the DSM-IV (*Diagnostic and Statistical Manual of Mental Disorders, Fourth Edition*). The PHQ-9 measures overall depression severity as well as the specific symptoms.

To measure quality of life (QoL), we used the WHOQOL-BREF. The WHOQOL-BREF is a self-report questionnaire that contains 26 items and addresses 4 QoL domains: physical health (7 items), psychological health (6 items), social relationships (3 items), and environment (8 items). Two other items measure overall QoL and general health.

During the intervention, self-report ratings of participants' confidence in managing the target anxiety situation were taken using a 6-point visual analogue scale at the following times: preintervention (beginning of session 1), end of session 2 (end of first day of intervention), beginning of session 3 (start of second intervention day), and at the end of session 4 (final session). The supporter was also asked to rate their perception of the participant's confidence at the same time points (ratings were taken in separate rooms for the participant and supporter and not shared). Ratings were from 0 (not at all confident) to 6 (very confident).

### Treatment procedures 

The treatment procedures were as follows: after a telephone discussion between a member of the research team with potential participants to establish whether their phobia was suitable for treatment, information sheets were sent to the participant and an initial home visit arranged. A phobia was judged as suitable if it could be reproduced and graded in the VR space. At this initial home visit, the participant was able to watch a video demonstration of the Blue Room intervention and have any questions answered. Written informed consent was obtained and initial discussion of the phobia to address and completion of baseline measures took place. A supporter, usually a family member, friend, or paid personal assistant was present at this initial visit and also gave written informed consent for their participation in the study.

During a second home visit, the participant and supporter met the state (NHS) registered qualified clinical psychologist delivering the intervention. At this visit, the therapist introduced the anxiety management strategies that would be used in the VRE sessions (e.g., scripted relaxation and breathing exercises/coping self-statements) as well as providing some psychoeducation in emotion recognition (including anxiety). The concept of a “feeling thermometer,” a visual scale to communicate intensity of anxiety, was also introduced to enable the participant to communicate the strength of their anxiety during the graded exposure sessions. The therapist also explored with the participant the possible steps in graded exposure in the VRE and the participant's treatment goal. These graded steps were used in the subsequent design briefs for the scene sent to the programmer from Third Eye Neurotech.

The first VRE session was 1–2 weeks after the second home visit, and this allowed time for participants to practice the relaxation techniques and for scene preparation. Participants and their supporters visited the Blue Room VRE on two separate occasions, receiving two 20- to 30-minute sessions on each visit with a break of 15 minutes between sessions. Visits were ∼1 week apart. During the four sessions, the participants, along with the therapist, worked through the agreed exposure hierarchies in relation to the phobia targeted. Participants moved up their hierarchy once they were able to comfortably manage their anxiety at each stage of exposure. Mastery was measured by self-report of sustained lowered anxiety level on the visual scale and verbal agreement that the participant was ready to move to the next level. The supporter(s) observed sessions via a video link in an adjacent room. This allowed supporters to observe the techniques the participant was practicing to cope with increasing levels of exposure. At the end of the fourth session, the therapist, participant, and supporter planned together the steps to real-life exposure.

A favorable ethical opinion was provided by the UK NHS Tyne and Wear South Ethics Committee (reference 12/NE/0018). The research sponsor was Northumberland, Tyne and Wear NHS Foundation Trust.

## Results and Lessons Learned

Table 1 shows information about each participant: their phobia, the impact on their daily living pretreatment (termed here “functional impairment”), the VRE scene designed for them, a description of their functional impairment in everyday life at 6 weeks and 6 months postintervention, and the change in target behavior ratings at 6 weeks and 6 months post-treatment, as rated by the blinded panel. Of the eight participants, five were classified as treatment responders (mean score of 3.0 or less as rated by the four-person rating panel) on the change in Target Situation Rating at 6 weeks and at 6 months postintervention. Of these five, four had a score of 1.0 or 1.25 at 6 months postintervention, indicating that the participant was able to function normally without any impact from the phobia. The five responders showed a pattern of increasing improvement with time as indicated by the improvement in target behavior scores from 6 weeks to 6 months follow-up, indicating a strengthening of the treatment effect over time. Three of the participants were nonresponders to treatment, each scoring 4.0 (equivocally improved and indicating no worsening of symptoms).

**Table 1.** Participant's Phobia/Fear and Associated Functional Impairment, Virtual Reality Scene Designed, and Outcomes Following Treatment (Functional Progress with Phobia/Fear and Target Behavior Scores)

*Participant*	*Situation*	*Functional impairment at baseline*	*VRE scene designed*	*Supporter and observers of sessions*	*Outcomes following the VRE treatment sessions; examples showing outcomes at 6 weeks and 6 months*	*Change in target behavior*	
						*6 Weeks*	*6 Months*
A	Open spaces	Unable to walk or drive through an area where there is an open landscape. Can have a panic attack if in these areas.	Participant and therapist are “in a car” and driving along a road that becomes increasingly open and without landmarks.	Friend	At 6 weeks, participant was able to drive on open roads on three separate occasions for ∼3 miles each time. At 6 months, participant had lost contact with the supporter and had not attempted to overcome fear due to a number of other difficult life events. Expressed a wish to have more sessions.	3.5 (Equivocally improved)	4.0 (Equivocally improved)
B	Walking through doorways	Unable to walk through any doorways alone and needs to walk behind someone. Anxiety is worse if the door is solid (no glass). The fear meant participant had to leave university.	A series of scenes where participant has to walk through a doorway to different rooms and increasingly opaque doors.	Parents and support worker	At 6 weeks, participant was progressing with going through more and more doorways with the support worker helping with this. At 6 months, able to go through every doorway alone. Has returned to university and has moved into university accommodation. Now able to go to the gym, swimming, and walking without support.	2.5 (Definitely improved)	1.0 (Normalized)
C	Spiders	Participant's phobia means many safety behaviors in place, e.g., tape around skirting boards, room sprayed with insect repellent every night, partner checking bedroom completely every night, light-colored wallpaper.	Living room and bedroom with increasing number of spiders and spiders disappearing under things (part of the phobia).	Mother, sister, and partner	Participant and partner moved to a new house shortly after the VR sessions. Has seen one spider in the new house. At 6 weeks postintervention, there were no safety behaviors utilized, i.e., no tape, no spraying of the bedroom, no checking at night, improved sleep. Partner said participant has been able to get up through the night and go downstairs alone. At 6 months postintervention, C was still not using any of the previous safety behaviors and reportedly not afraid when sees a spider.	1.5 (Markedly improved)	1.0 (Normalized)
D	Babies/pram	At any sight of a baby or pram, participant will become agitated and try and escape the situation. The family are worried regarding safety as D will bolt into the road when he sees a baby or pram. This has led to an increasing reluctance to go out by both the participant and family. Unable to be in the same house as baby nephew.	Scene with prams and babies in increasing numbers and making increasing amounts of noise.	Parents, brother, and support worker	At 6 weeks, participant now able to travel on a bus for the first time in 10 years. Can now walk past prams happily in a busy city center, whereas would have bolted before. Able to sit in a cafe for 20 minutes without being hypervigilant for prams/babies. Has managed a variety of shopping and outing situations where there were lots of babies. Able to talk to baby nephew via FaceTime (previously hid all pictures of him in the house). Able to self-calm much more now when gets agitated. At 6 months post-treatment, there had been a few setbacks, including significant distress while on a plane where there was a crying baby. However, overall, the family felt that there was improvement and D was able to meet nephew and spend some time with him.	3.0 (Definitely improved)	4.0 (Equivocally improved)
E	Making requests	Participant unable to make requests or ask for help, particularly if these requests are related to help needs with physical disability.	A series of scenes where had to ask for help from virtual people for different reasons, e.g., directions, open questions—some virtual people were helpful and some not.	Mother	At 6 weeks after the intervention, the participant was able to: Ask for food to be cut up in restaurant Asked a taxi driver to help with carrying shopping bags. Asked for help in a supermarket Asked someone to come for coffee At 6 months postintervention, E has maintained confidence in making requests and now persists if does not get the response needed, e.g., with medical matters. Has applied for three jobs since treatment sessions and has had interviews for two of them. Is now able to ask colleagues where they volunteer for support with physical disability.	1.5 (Markedly improved)	1.0 (Normalized)
F	Pigeons	Long-standing fear of pigeons. Participant has a number of animal phobias, but the pigeon phobia is preventing visits to town centers here and abroad or eating outside.	Village square scene with increasing numbers of pigeons able to fly in and out of the scene. Can move up close to the pigeons.	Mother	At 6 weeks postintervention, the participant was able to be near pigeons while in the local town. Managed to sit in an outdoor cafe where there were pigeons nearby and eat. At 6 months, F has maintained confidence in dealing with pigeons and said had made improvements that previous CBT alone for animal phobia had not been able to deliver.	2.75 (Definitely improved)	1.25 (Normalized)
G	Insects/flies	Participant can have panic attacks if an insect/fly is near in the house and will be unsettled for a long time afterward. Cannot sit outside or eat outside because of the fear.	A street scene, a back garden, and a fast food scene. Flying insects appear in each of these scenes.	Parents	The participant set target to be able to eat a meal outside and had achieved this at 6 weeks postintervention. Has been able to sit in the garden for increasing amounts of time. At 6 months postintervention, able to catch insects in a glass and put them outside. Has been able to eat outside in the garden and at favorite fast food restaurant (target scene). Although very able, G often struggles to get ideas into words but has “found his courage” and is able to not run away now when afraid.	3.5 (Equivocally improved)	2.0 (Markedly improved)
H	Crowded buses	Unable to travel by bus and has given up college because of this. Recently tried to walk to bus stop with support worker, but anxiety became overwhelming. Anxiety is increased if bus is crowded and if windows are steamed up.	Scene with long walk to bus stop and bus which arrives with increasing numbers of people. Can get on bus and windows gradually steam up and increasing background chatter as bus fills up.	Mother and support worker	At 6 weeks postintervention, H had begun walking to the bus stop with support worker (could not do this before). They continued to practice this until H was able to manage anxiety. At 6 months postintervention, H had been on the bus with either mother or support worker numerous times with journeys of ∼40 minutes. Next aim is to progress to journey alone.	3.5 (Equivocally improved)	4.0 (Equivocally improved)

CBT, cognitive behavioral therapy; VR, virtual reality; VRE, virtual reality environment.

**FIG. 1.**
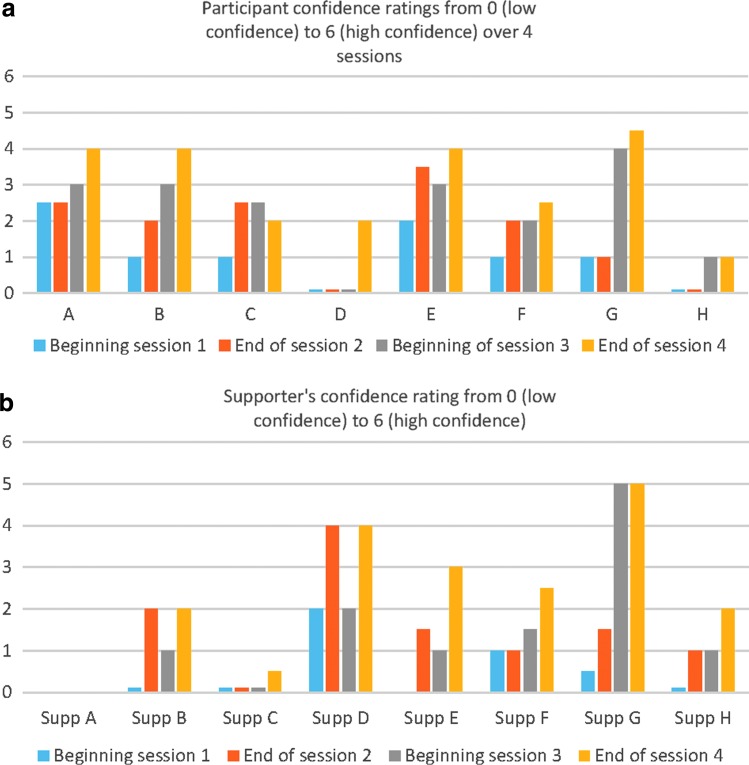
**(a)** Participant confidence ratings at tackling the target situation at four time points. **(b)** Supporter confidence ratings at tackling the target situation at four time points. Supporter A data missing as unable to rate as she had not seen participant in the real-life situation.

Figure 1a and b shows confidence ratings in tackling the phobia as rated by the participant and the supporter from the beginning of session 1 until the end of treatment at session 4. Overall, for both participants and responders, confidence ratings increased from the beginning of session 1 to the end of session 4, although the magnitude of the increase varied considerably.

Table 2 shows the means and standard deviations at an individual and group level at preintervention, 6 weeks postintervention, and 6 months postintervention for the BAI, PHQ-9, and GAD-7. Table 3 shows the group means and standard deviations for four domains of the WHOQOL-BREF: physical, psychological, social, and environment. The reliable and clinically significant change indices were calculated for the GAD-7, PHQ-9, and BAI. We found no consistent pattern of reliable or observable changes on the standardized measures (data not shown).

**Table 2.** Mean Group Scores and Individual Scores for the Beck Anxiety Inventory, Patient Health Questionnaire-9, and Generalized Anxiety Disorder-7 at Preintervention, 6 Weeks Postintervention, and 6 Months Postintervention

	*PHQ-9*			*BAI*			*GAD-7*		
	*Preintervention*	*6 Weeks postintervention*	*6 Months postintervention*	*Preintervention*	*6 Weeks postintervention*	*6 Months postintervention*	*Preintervention*	*6 Weeks postintervention*	*6 Months postintervention*
Group mean (SD)	10.8 (8.1)	9.4 (8.4)	10.0 (8.0)	26.9 (16.4)	26.3 (21.9)	28.4 (22.2)	9.5 (4.8)	9.8 (7.04)	9.5 (6.2)
Participant									
A	24	24	24	54	63	63	18	21	21
B	2	2	2	16	14	11	4	4	2
C	13	8	15	26	19	21	13	16	7
D	0	0	1	14	N/A	N/A	6	5	4
E	6	6	5	13	9	4	6	4	5
F	19	15	14	27	18	34	11	7	12
G	10	3	5	15	9	14	12	4	11
H	12	17	14	50	52	52	6	17	14

BAI, Beck Anxiety Inventory; GAD-7, Generalized Anxiety Disorder-7; N/A, not available; PHQ-9, Patient Health Questionnaire-9.

**Table 3.** Mean Scores for WHOQOL-BREF Subscales

*Time point*	*Mean physical*	*Mean psychological*	*Mean social*	*Mean environment*
Preintervention	39.29 (14.91)	46.35 (15.98)	41.67 (16.06)	53.13 (14.94)
6 Weeks postintervention	31.12 (15.93)	45.83 (15.96)	47.02 (23.41)	57.59 (22.09)
6 Months postintervention	40.6 (16.19)	45.83 (14.26)	51.04 (19.64)	59.77 (19.87)

Higher scores in each subscale indicate higher quality of life.

Three case studies are presented in detail below as exemplars of different treatment journeys and responses.

### Case study 1

Participant B was a 20 year old with a fear of walking through doorways. At the start of the study, the participant lived with parents.

At baseline, B was unable to walk through any doorway alone and had to have a known person walk through the doorway in front. B's anxiety increased the more opaque the entrance doors—solid doors being the most difficult. The fear was having a significant impact on functioning and was a contributing reason to withdrawing from university. B reported receiving CBT in a clinic setting previously regarding this phobia, but anxiety levels had not reduced.

For intervention sessions, a series of VRE scenes were designed where B had to virtually walk through a doorway into rooms with different interiors and through increasingly solid doors. The therapist was able to establish and challenge B's thoughts regarding walking through doorways and help B find strategies to deal with different situations and occurrences, for example, not knowing where to sit when entering a room; what to say if someone spoke. B's parents and a support worker observed the sessions. Having observed the techniques used by the therapist in the VRE, the support worker was then able to use these to help B practice walking through doorways in real life. This was consolidated when B was on outings with family.

B and the support worker set various real-life targets of doorways to go through. Gradually, B managed to go through more doorways and built up to entering rooms and buildings without support. At 6 months postintervention, B was able to go through all doorways independently. B was able to go to the gym and swimming pool without support and had returned to university and was attending lectures. B moved from the family home to shared university accommodation. Participant described as more aware of situations that might generate anxiety and being able to utilize strategies to manage these situations, for example, not arriving too early for lectures, and/or recognizing times when susceptible to anxiety, for example, around examination time.

### Case study 2

A was a 50 year old who lives independently, with a fear of open spaces.

A was unable to walk or drive through an area where there was an open landscape. For example, if walking in the countryside and the landscape became very open, participant A could have a panic attack. If driving or cycling or walking, A would have to prepare thoroughly beforehand by making detailed travel plans using maps to avoid open roads or motorways. This took up a great deal of time before a journey (some days) and the participant carried maps to feel safe.

The scene designed after discussion with A was a virtual car in which A was the driver traveling along a road increasingly devoid of landmarks. During the sessions, A was able to articulate how anxiety increased as the VRE scene changed. While in the VRE, A became aware that anxiety began to rise as the landmarks on the virtual road started to disappear (something A had not been able to describe before the VRE sessions); A “dissociates” at this point. The therapist was able to suggest a technique to help A focus back on managing anxiety; this involved A imagining painting the landscape. This helped A work toward reducing anxiety. The supporter that A chose (and who observed the VRE sessions) was a friend from a local support group.

Following VRE sessions, A had a number of difficult life events that reduced capacity to practice real-life exposure and consolidate new learning. Participant A managed to drive on open motorway on three occasions for several miles but was unable to continue with the practice. A's supporter was also no longer able to help. At both 6 weeks and 6 months after the intervention, A reported still feeling anxious in open spaces and was often unable to tolerate them. A commented more sessions might have helped and a different supporter.

### Case study 3

F was a 22 year old with a fear of pigeons and lives with parents.

F had a number of animal fears, but the pigeon phobia was preventing regularly visiting the local city and other cities. If travel was necessary, F became very anxious and undertook detailed research to identify areas where pigeons were likely to be. After agreeing the target situation, the VR scene designed was a village square with tables outside and increasing numbers of pigeons that could fly into and out of the scene. The therapist explored F's thoughts around pigeons during the VRE session. During VRE sessions, F learnt to connect the hypervigilance around pigeons to the increasing anxiety experienced. F became aware in the VRE that when anxious and then able to “move back” from the situation slightly, then F was able to reduce anxiety and prepare to move toward the virtual pigeons. The supporter was F's mother. F was able to negotiate with her about using a similar strategy in real-life situations with pigeons.

F gradually increased exposure to pigeons in real life, starting with watching them in the garden and progressing to eating outside near pigeons and using the relaxation and coping self-statements F had practiced in the Blue Room VRE. At 6 months after the intervention, F was able to walk past pigeons when in the local city. F was also able to sit at outdoor cafes when there were pigeons nearby and no longer avoided cities where there were pigeons. F reported that that the VRE experience had been of greater benefit than previous traditional clinic-based CBT received for a dog phobia and that in the VRE participant F experienced more realistic levels of anxiety (and subsequent practice at reduction) than in the clinic setting.

The results described above show us that this method of VR exposure may be an effective intervention for anxiety in autistic adults. We learned that computer-generated scenes are acceptable and believable for autistic adults and that four sessions of intervention are feasible to deliver and attend. This treatment can result in real-life improvements for autistic adults. The role of the supporter in this study is an important one, and in future studies, we will explore how best to support the person in this role. This treatment is effective with a number of fears/phobias; in the future, larger studies we will investigate response to treatment for a greater number of fears/phobias.

## Discussion

This is the first report of a CBT and immersive VRE intervention for adults on the autism spectrum experiencing phobias. We demonstrated that the treatment package of graded exposure and anxiety management strategies in the VRE is feasible to deliver and acceptable, with all participants completing all four sessions. In line with aim 1 and aim 2 of the study, we have demonstrated that autistic adults engage with the computer-generated VRE scenes in a way that allows them to approach and remain in the anxiety-provoking situation and we had retention and participation for all sessions. Our third aim was to investigate if four sessions were appropriate and this number of sessions resulted in change for some participants. We will investigate further in future studies if this number should be increased.

For all participants, phobia was having a major impact on their lives (e.g., preventing attendance at university, preventing travel, and/or involving extensive safety rituals that limited their participation in everyday activities). Five of the eight adults improved in their ability to tackle their real-life phobia, and four adults were able to function in everyday life without any impact from their phobia. The results from this small study show similar levels of improvement to the use of CBT alone. Based on a meta-analysis of 42 placebo-controlled trials for anxiety disorders in the general population, Carpenter et al.^20^ reported Hedges' g = 0.56 (*N* = 2843, 95% confidence interval = 0.44–0.69, *p* < 0.0001) and concluded that “CBT is a moderately efficacious treatment for anxiety disorders with variability in outcomes according to disorder.”^20^

Three people showed less improvement during the VRE intervention. Adult A reported being unable to put the strategies learnt in the VRE into practice due to personal circumstances. In a postintervention interview, A said that further sessions would have been useful (see the Case Study 2 section). Adult D showed an initial high level of improvement in anxiety management, but this reduced over time, particularly over the summer holidays from college when their routine changed. D's parents also indicated that they would have liked further sessions. Adult H did not meet the criteria for responder. However, at the 6-month follow-up, H was making progress toward the goal to travel on a bus independently.

The standardized questionnaires used did not consistently show any changes in progress following the intervention; this was in keeping with the previously reported children's study.^12^ This may be due to the limitations of these measures to detect changes in anxiety related to a phobia in an autism spectrum population. No measure for phobia in autistic people exists currently (see aim 4).

The relationship of the supporter to the participant varied across the study and included parents, friends, partners, and support workers. In three cases, the support worker attended with a family member. Postintervention interviews with supporters indicated that while they were clear about their general role, more specific guidance about the role would have been valuable, including guidance on how to best support the participant to tackle their real-life anxiety target.

While the recognized intervention for phobia in the general population is manualized CBT, there is growing evidence that using a VRE to facilitate graded exposure has a place in treatment.^21,22^ Our data indicate that the adaptations we have implemented by using the VRE technique may confer a number of benefits for some autistic adults. They allow exposure at a level of anxiety that feels safe and is not overwhelming; at the same time, VR is real enough to induce anxiety at a level for habituation to occur through using anxiety management strategies to stay in the situation. The visual nature of learning in the VRE may allow the participant to access their feared situation without becoming overwhelmed by other sensory modalities; the controllable nature of the VRE may enable the participants to be more able to interact with the therapist and practice the techniques they are learning. Exposure to the same point on the anxiety hierarchy can be undertaken a number of times to enable the participant to develop confidence in, and mastery of, the anxiety management strategies being practiced and experience a reduction in anxious affect at one level before moving up the hierarchy to a greater level of challenge. The treatment protocol also enables a supporter to watch the techniques being developed in the VRE, via video link. This opportunity would be difficult to create in traditional CBT settings but may be critical to support the generalization of the techniques to real-world settings. Observation of the anxiety reduction techniques developed in the VRE enabled the supporter to scaffold and support their use during real-world exposure. However, the ways in which supporters did this were not specifically explored. Likewise, it is not known whether changes are due to learning about or habituation to specific elements of the situation, a greater understanding of the nature and timecourse of acute anxiety responses, development of appropriate anxiety control strategies, confidence in using them, or increased self-efficacy.^23^

This study tested a focused intervention for autistic adults who were experiencing a fear or phobia that was having a significant impact on their lives. The study builds on the demonstrated positive effects of this approach with autistic children^12,13^ and provides initial observations of the VR intervention delivered to adults. In line with aim 5, we have shown that there are improvement in outcomes following the intervention for some adults. It will be important in the future to recruit a larger group of adults with a range of life-limiting situational phobias and investigate the magnitude of treatment effect. The inclusion of a control group and a blinded researcher to collect the target behavior vignettes will be important in future studies. The control group could be a delayed treatment group of adults with autism and phobia, people randomized to usual care, or a group of adults with autism who are receiving a typical CBT package of treatment for phobia. Feedback from participants indicated that the role of the supporter needs to be more clearly defined, and the limits of the role stated for both the supporter and participant.

### Limitations and future directions

The limitations to this study are the small sample size, which precluded statistical analysis. Future studies should have a blinded outcome assessor as a further limitation to the current study is that the researcher writing the vignettes was not blind to treatment status.

Further research investigation will be undertaken through evaluation of effectiveness in clinical practice or a randomized controlled trial. Future research will aim to identify the mechanisms leading to effective treatment including which adaptations are required, whether fewer or more sessions are effective for some people, or whether subsequent booster sessions are helpful for partial, or nonresponders will be important, as will understanding how an individual's autism spectrum characteristics and phobia type affect the likelihood of treatment success.
